# Supplementary material for: Sensory trial of camel milk powder among pastoralist communities of the Somali Region, Ethiopia
Source: PLoS One. 2025 Oct 30;20(10):e0333358. doi: 10.1371/journal.pone.0333358 (PMC12574850; doi:10.1371/journal.pone.0333358)
Supplement: S2 File — (DOCX) [file pone.0333358.s002.docx]

**Questionnaire** (Version A)

1. **Place of Residence Rural ☐**

**Urban ☐**

1. **Gender Male ☐**

**Female ☐**

1. **Age ________ in Years**
2. **How do you like this milk?**

|  | 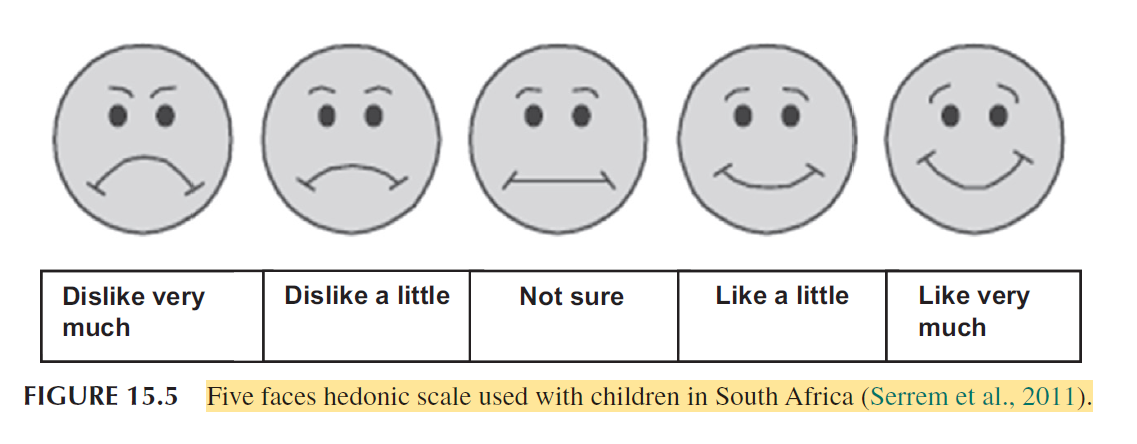 | 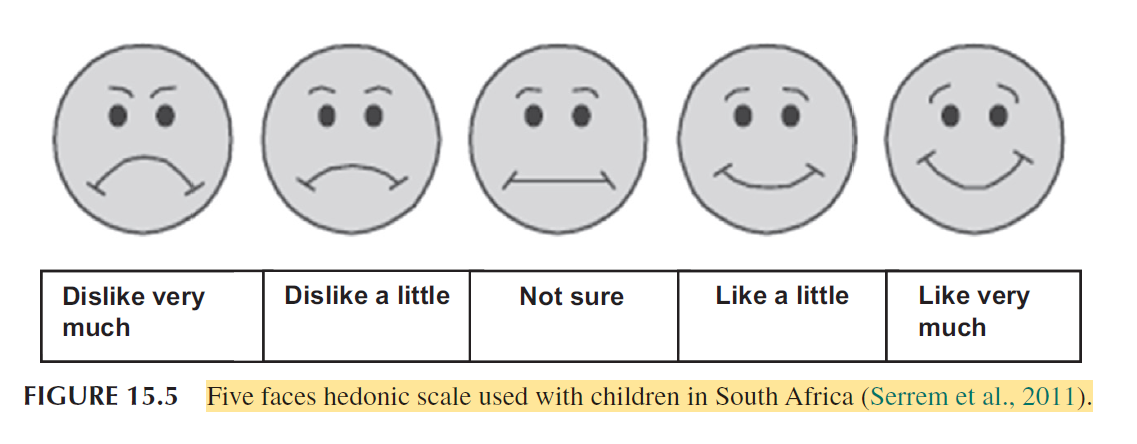 | 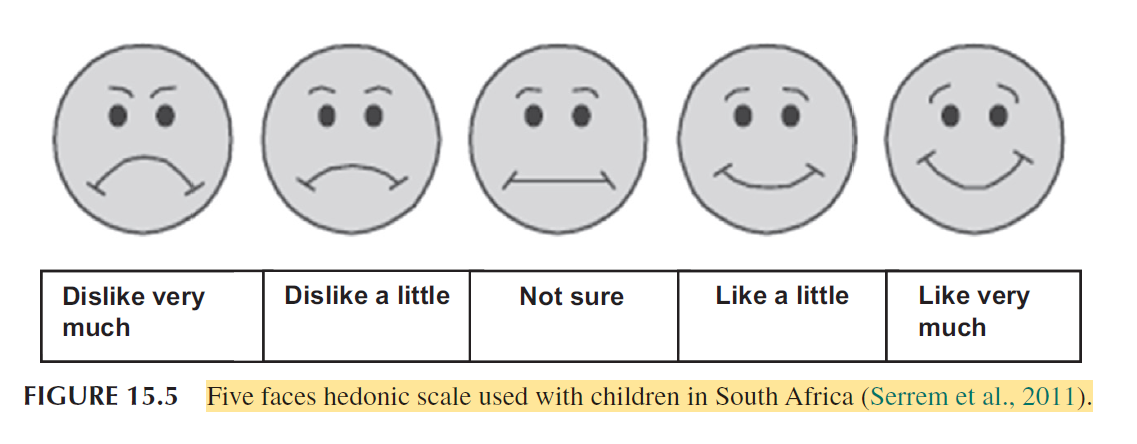 | 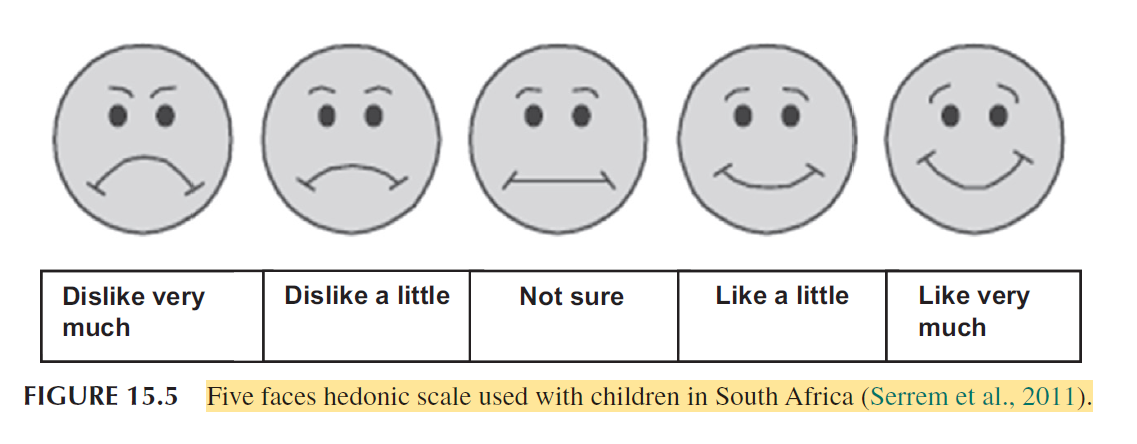 | 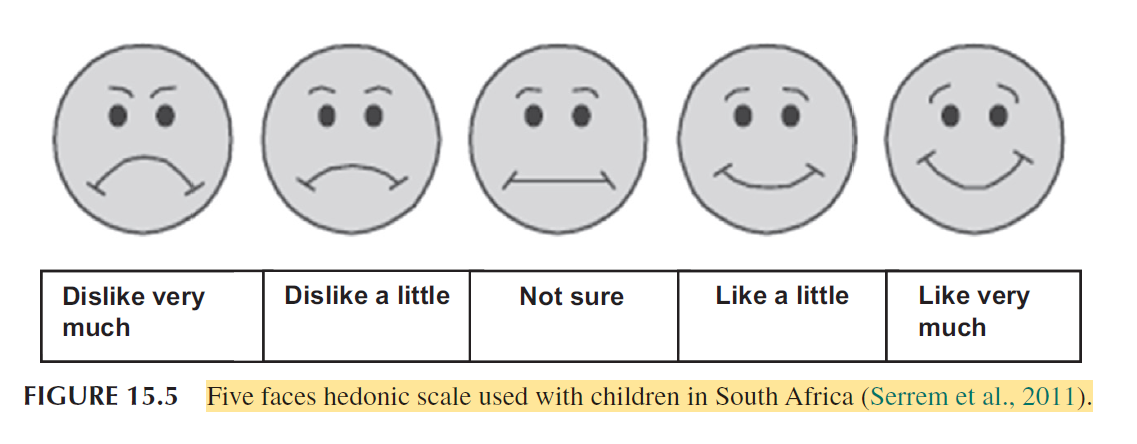 |
| --- | --- | --- | --- | --- | --- |
|  | ☐ | ☐ | ☐ | ☐ | ☐ |
|  | ☐ | ☐ | ☐ | ☐ | ☐ |

1. **How much would you be prepared to spend for each milk?**

|  | 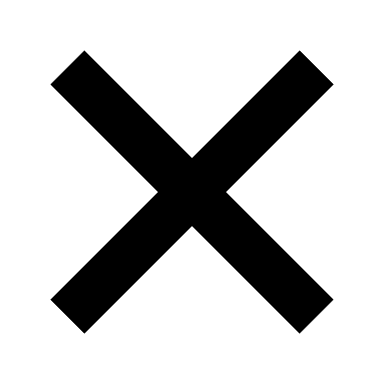 | 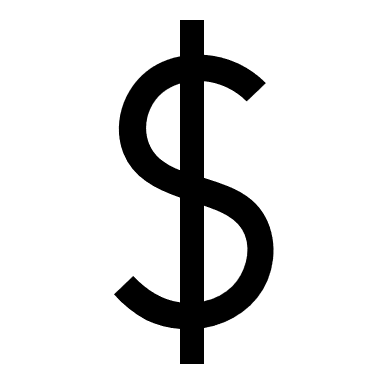 | 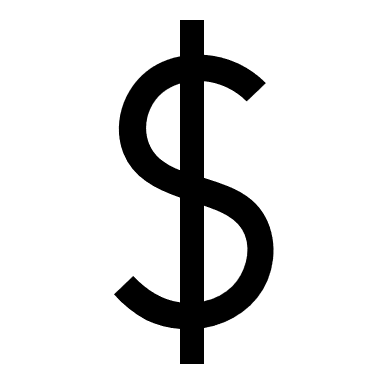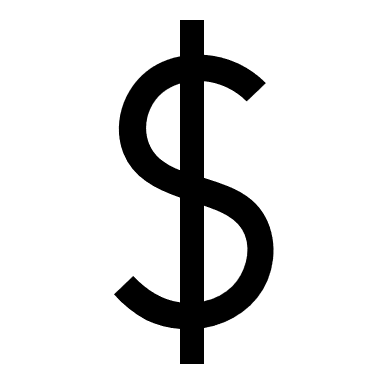 | 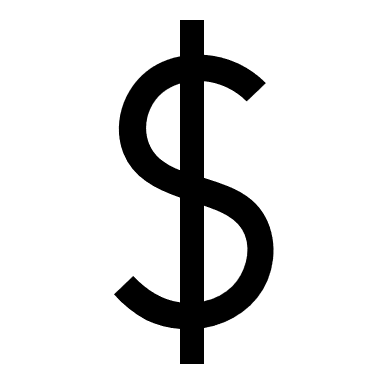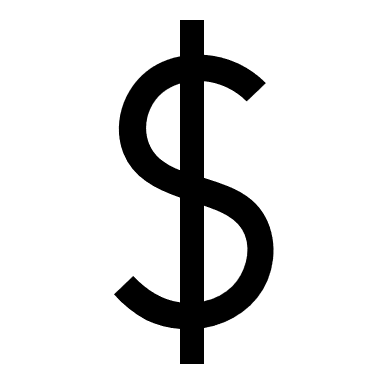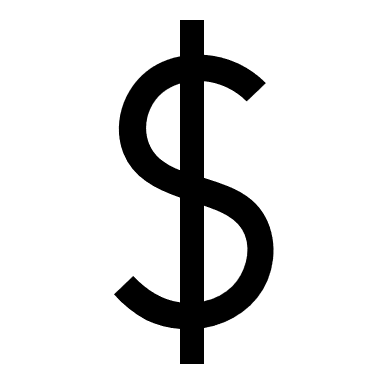 |
| --- | --- | --- | --- | --- |
|  | ☐ | ☐ | ☐ | ☐ |
|  | ☐ | ☐ | ☐ | ☐ |

1. **Which of the milks did you prefer?**

|  | **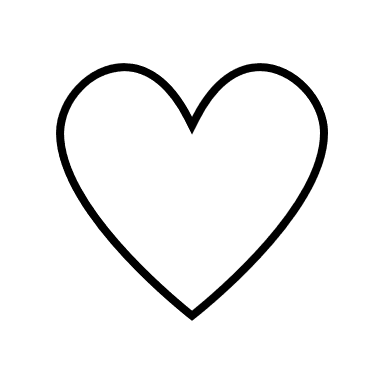** |
| --- | --- |
|  | ☐ |
|  | ☐ |

**Questionnaire** (Version B)

1. **Place of Residence Rural ☐**

**Urban ☐**

1. **Gender Male ☐**

**Female ☐**

1. **Age ________ in Years**
2. **How do you like this milk?**

|  | 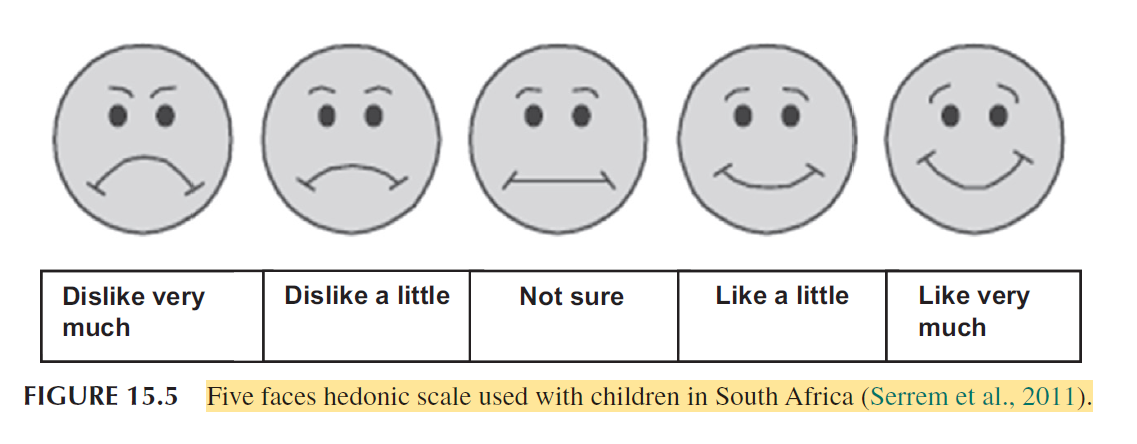 | 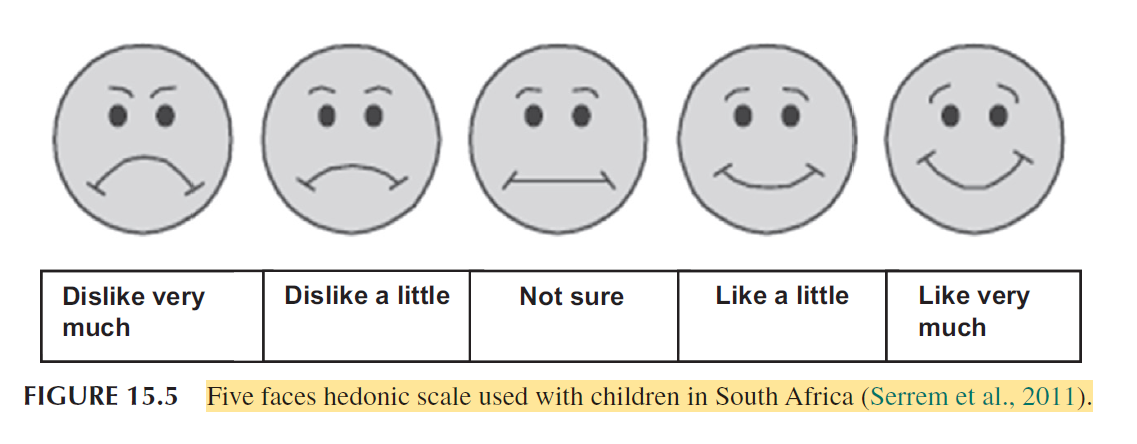 | 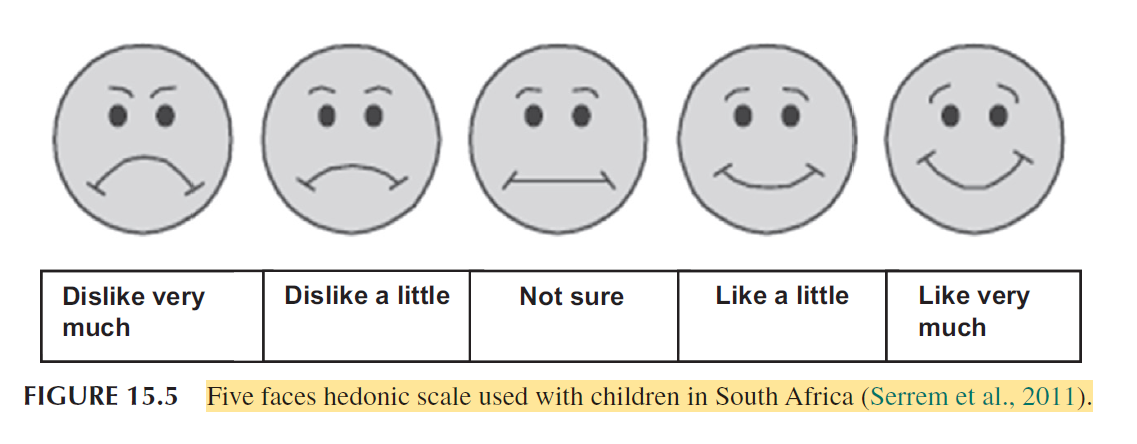 | 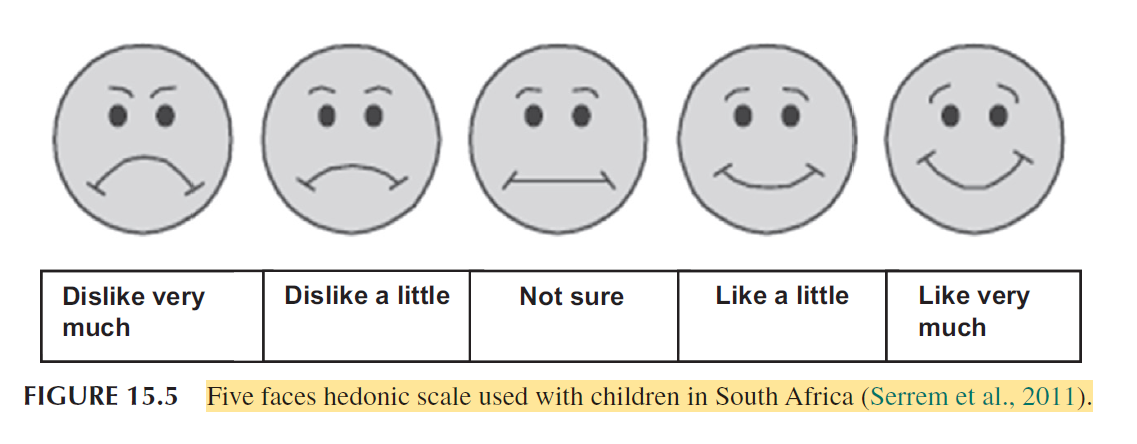 | 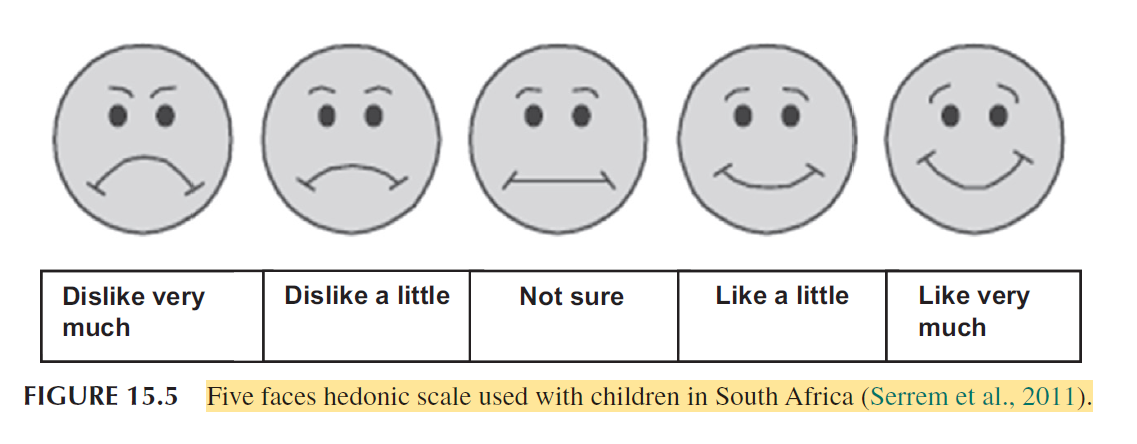 |
| --- | --- | --- | --- | --- | --- |
|  | ☐ | ☐ | ☐ | ☐ | ☐ |
|  | ☐ | ☐ | ☐ | ☐ | ☐ |

1. **How much would you be prepared to spend for each milk?**

|  | 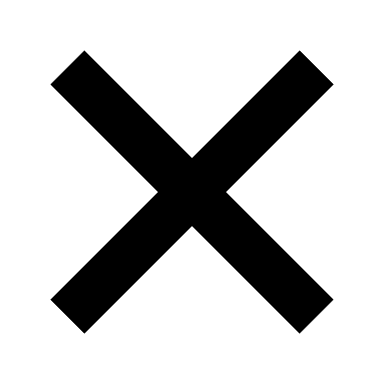 | 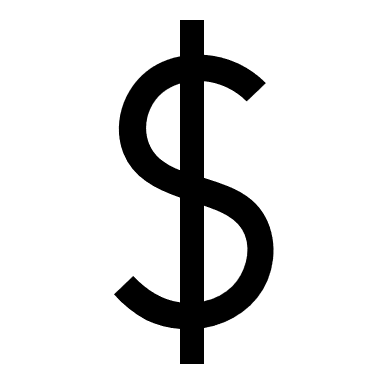 | 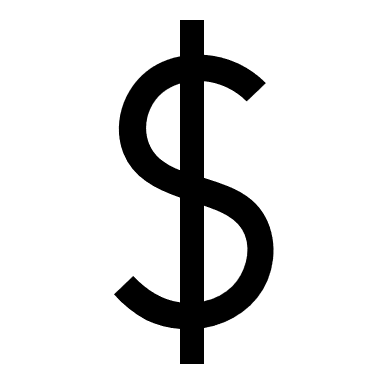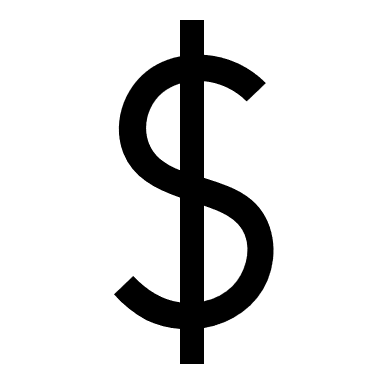 | 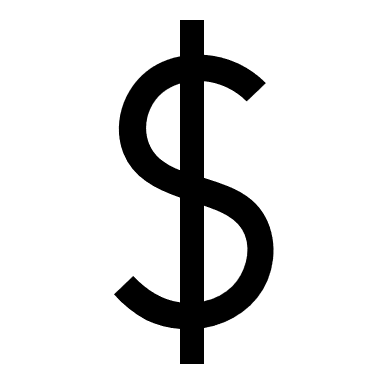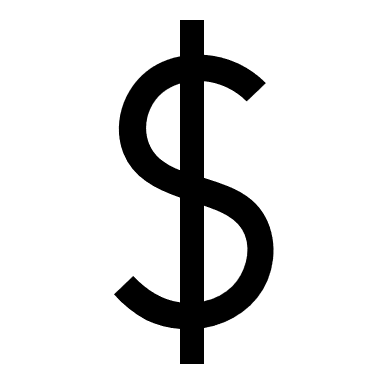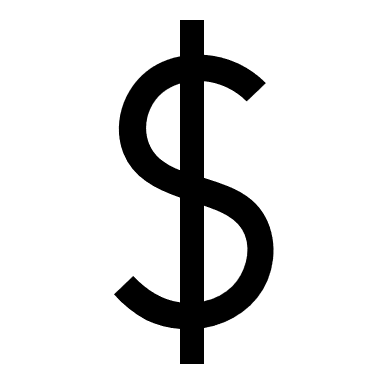 |
| --- | --- | --- | --- | --- |
|  | ☐ | ☐ | ☐ | ☐ |
|  | ☐ | ☐ | ☐ | ☐ |

1. **Which of the milks did you prefer?**

|  | **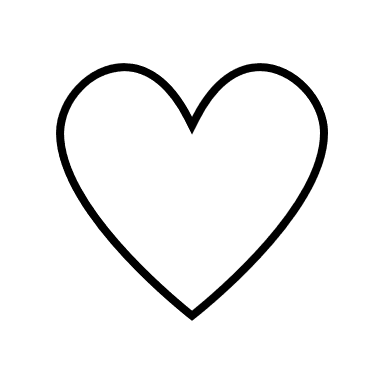** |
| --- | --- |
|  | ☐ |
|  | ☐ |
