# Supplementary material for: Sensory trial of camel milk powder among pastoralist communities of the Somali Region, Ethiopia
Source: PLoS One. 2025 Oct 30;20(10):e0333358. doi: 10.1371/journal.pone.0333358 (PMC12574850; doi:10.1371/journal.pone.0333358)
Supplement: Attachment — Submitted filename: PONE-D-25-31077 revised.pdf [file pone.0333358.s004.pdf]

PLOS ONE

SENSORY TRIAL OF CAMEL MILK POWDER AMONG PASTORALIST COMMUNITIES OF THE SOMALI REGION, ETHIOPIA

--Manuscript Draft--

**Manuscript Number:**	PONE-D-25-31077
**Article Type:**	Research Article
**Full Title:**	SENSORY TRIAL OF CAMEL MILK POWDER AMONG PASTORALIST COMMUNITIES OF THE SOMALI REGION, ETHIOPIA
**Short Title:**	SENSORY TRIAL OF CAMEL MILK POWDER AMONG PASTORALIST COMMUNITIES
**Corresponding Author:**	Ahmed Mohammed Ibrahim<br>Jigjiga University<br>jigjiga, Somali ETHIOPIA
**Keywords:**	Sensory Trial, Camel Milk Powder, Pastoralist Communities, Somali Region, Ethiopia
**Abstract:**	ABSTRACT<br>Introduction: Camel milk is a vital source of nutrition for people living in many arid and semi-arid regions. Camel milk powder offers a valuable opportunity for the dairy industry to develop and launch innovative products in the milk and dairy market.<br>Objective: To evaluate the sensory preference of powdered camel milk compared to fresh milk among pastoralist communities in the Somali Region of Ethiopia.<br>Methods: - Sensoric cross over experiment study was conducted among randomly selected 242 pastoralist communities in Somali Region. Data were collected using structured questionnaires. The sensory trial was single-blinded; participants did not know whether they tasted fresh or powdered camel milk. The sequence was randomized, assigning either fresh/powdered camel milk or powdered/fresh camel milk to each participant. Data was summarized, such as mean, median, frequency, and percentage, and was presented using charts and tables. Bi-variable and Multivariable binary logistic regression identified variables associated with the outcome. Statistical associations were assessed using odds ratios and 95% confidence intervals, with significance declared at a P-value < 0.05. A t-test was used to compare milk spending and milk liking levels between powdered and fresh camel milk.<br>Results: In this study, 167 (69%; 95% CI: 62.9%–74.5%) preferred fresh camel milk, while 75 (31%; 95% CI: 25.5%–37.1%) preferred reconstituted powdered milk among pastoralists in the Somali Region. Factors significantly associated with preference of reconstituted powdered camel milk included being an urban pastoralist (AOR = 2.02; 95% CI: 1.30, 3.16) and being female (AOR = 1.94; 95% CI: 1.25, 3.01). There is no statistically significant mean difference between fresh camel milk and powdered camel milk regarding willingness to pay.<br>Conclusion: Although most preferred fresh milk, the mean rating of powder was still high.<br>Powdered milk might be a good alternative in settings where a cool chain is difficult to maintain and shelf life is an issue. Factors such as place of residence and sex of pastoralist significantly influence preference of reconstituted powdered milk. There is no significant difference in willingness to pay between fresh and powdered milk. Focusing on nutritional advantages, safety, and convenience regarding powdered camel milk is crucial for rural and male pastoralists' adoption. Promotional efforts should increase accessibility and practicality without reducing cost.
**Order of Authors:**	Ahmed Mohammed Ibrahim
	Seid Mohammed Ali
	Yahya Maidane Osman
	Fathiya Budul Ismail
	Mohamed Omar Osman
	Mukhtar Harir Hussein

Powered by Editorial Manager® and ProduXion Manager® from Aries Systems Corporation

	Abdifatah Muktar Muhummed
	Ramadan Budul Yusuf
	Pauline Rouchon
	Jan Hattendorf
	Rea Tschopp
	Raymond Place
	Jakob Zinsstag
**Additional Information:**	
**Question**	**Response**
**Financial Disclosure**<br><br>Enter a financial disclosure statement that describes the sources of funding for the work included in this submission. Review the submission guidelines for detailed requirements. View published research articles from *PLOS ONE* for specific examples.<br><br>This statement is required for submission and **will appear in the published article** if the submission is accepted. Please make sure it is accurate.<br><br>**Funded studies**<br>Enter a statement with the following details:<br>• Initials of the authors who received each award<br>• Grant numbers awarded to each author<br>• The full name of each funder<br>• URL of each funder website<br>• Did the sponsors or funders play any role in the study design, data collection and analysis, decision to publish, or preparation of the manuscript?<br><br>Did you receive funding for this work?	The author(s) received no specific funding for this work.
**Competing Interests**<br><br>Use the instructions below to enter a competing interest statement for this submission. On behalf of all authors, disclose any competing interests that could be perceived to bias this work—acknowledging all financial support and any other relevant financial or non-	The authors have declared that no competing interests exist

financial competing interests.<br><br>This statement is required for submission and **will appear in the published article** if the submission is accepted. Please make sure it is accurate and that any funding sources listed in your Funding Information later in the submission form are also declared in your Financial Disclosure statement.<br><br>View published research articles from *PLOS ONE* for specific examples.<br><br>**NO authors have competing interests**<br><br>Enter: *The authors have declared that no competing interests exist.*<br><br>**Authors with competing interests**<br><br>Enter competing interest details beginning with this statement:<br><br>*I have read the journal's policy and the authors of this manuscript have the following competing interests: [insert competing interests here]*<br><br>* typeset	
**Ethics Statement**<br><br>Enter an ethics statement for this submission. This statement is required if the study involved:<br><br>• Human participants<br>• Human specimens or tissue<br>• Vertebrate animals or cephalopods<br>• Vertebrate embryos or tissues<br>• Field research<br><br>Write "N/A" if the submission does not require an ethics statement.<br><br>General guidance is provided below. Consult the submission guidelines for	Ethical was reviewed and approved by the Jigjiga University Research Ethics Review Committee (Approval No. JJU-RERC/002/2025). An official permission letter was also submitted to the relevant administrative offices in the Shebelle and Fafan Zones. Informed, voluntary, written, and signed consent was obtained from the pastoralist communities after the purpose and potential benefits of the study were clearly explained

detailed instructions. **Make sure that all information entered here is included in the Methods section of the manuscript.**<br><br>**Format for specific study types**<br><br>**Human Subject Research (involving human participants and/or tissue)**<br>• Give the name of the institutional review board or ethics committee that approved the study<br>• Include the approval number and/or a statement indicating approval of this research<br>• Indicate the form of consent obtained (written/oral) or the reason that consent was not obtained (e.g. the data were analyzed anonymously)<br><br>**Animal Research (involving vertebrate animals, embryos or tissues)**<br>• Provide the name of the Institutional Animal Care and Use Committee (IACUC) or other relevant ethics board that reviewed the study protocol, and indicate whether they approved this research or granted a formal waiver of ethical approval<br>• Include an approval number if one was obtained<br>• If the study involved *non-human primates*, add *additional details* about animal welfare and steps taken to ameliorate suffering<br>• If anesthesia, euthanasia, or any kind of animal sacrifice is part of the study, include briefly which substances and/or methods were applied<br><br>**Field Research**<br><br>Include the following details if this study involves the collection of plant, animal, or other materials from a natural setting:<br>• Field permit number<br>• Name of the institution or relevant body that granted permission	
**Data Availability**<br><br>Authors are required to make all data underlying the findings described fully available, without restriction, and from the time of publication. PLOS allows rare	Yes - all data are fully available without restriction

exceptions to address legal and ethical concerns. See the PLOS Data Policy and FAQ for detailed information.<br><br>A Data Availability Statement describing where the data can be found is required at submission. Your answers to this question constitute the Data Availability Statement and **will be published in the article**, if accepted.<br><br>**Important:** Stating 'data available on request from the author' is not sufficient. If your data are only available upon request, select 'No' for the first question and explain your exceptional situation in the text box.<br><br>Do the authors confirm that all data underlying the findings described in their manuscript are fully available without restriction?	
**Describe where the data may be found in full sentences. If you are copying our sample text, replace any instances of XXX with the appropriate details.**<br><br>• If the data are **held or will be held in a public repository**, include URLs, accession numbers or DOIs. If this information will only be available after acceptance, indicate this by ticking the box below. For example: *All XXX files are available from the XXX database (accession number(s) XXX, XXX.*).<br>• If the data are all contained **within the manuscript and/or Supporting Information files**, enter the following: *All relevant data are within the manuscript and its Supporting Information files.*<br>• If neither of these applies but you are able to provide **details of access elsewhere**, with or without limitations, please do so. For example:<br><br>*Data cannot be shared publicly because of [XXX]. Data are available from the XXX Institutional Data Access / Ethics Committee (contact via XXX) for researchers who meet the criteria for*	XXX

access to confidential data.<br><br>*The data underlying the results presented in the study are available from (include the name of the third party and contact information or URL).*<br>• This text is appropriate if the data are owned by a third party and authors do not have permission to share the data.<br><br>* typeset	
Additional data availability information:	

Powered by Editorial Manager® and ProduXion Manager® from Aries Systems Corporation

Manuscript Click here to access/download;Manuscript;MANUSCRIPT.docx

SENSORY TRIAL OF CAMEL MILK POWDER AMONG PASTORALIST COMMUNITIES OF THE SOMALI REGION, ETHIOPIA

Ahmed Mohammed Ibrahim[1,2, 3 *], Seid Mohammed Ali [1,2 3], Yahya Maidane Osman[3], Fathiya Budul Ismail[3], Mohamed Omar Osman[3, 7], Pauline Rouchon[4], Mukhtar Harir Hussein[1,2], Abdifatah Muktar Muhummed[3, 6], Ramadan Budul Yusuf[3], Raymond Place[4], Jan Hattendorf[1,2], Rea Tschopp[1, 2, 5] and Jakob Zinsstag[1,2].

1[Swiss Tropical and Public Health Institute, Kreuzstrasse 2, 4123, Allschwil, Switzerland]
2[University of Basel, Petersplatz 1, 4003, Basel, Switzerland].
3[Jigjiga University, Jigjiga, Ethiopia].
4[School of Agricultural, Forest and Food Sciences, Bern University of Applied Sciences (BFH-HAFL), Zollikofen, Switzerland].
5[Armauer hansen research institute].
6[Faculty of Medicine and Biology, University of Lausanne]
7[College of Medicine and Health Sciences, Department of Public Health, Hawassa University, Hawassa, Ethiopia]

Correspondence: *[Ahmed Mohammed Ibrahim]
[P.O.BOX: 1020, Jigjiga, Ethiopia]
Tel [+251912423271]
Fax [+251 (0) 25 775 5976]
Email:ahmey114baba@gmail.com/ahmedmohammed@jju.edu.et/ahmed.ibrahim@swisstph.ch/ahmed.ibrahim@unibas.ch.

ABSTRACT

Introduction: Camel milk is a vital source of nutrition for people living in many arid and semi-arid regions. Camel milk powder offers a valuable opportunity for the dairy industry to develop and launch innovative products in the milk and dairy market.

Objective: To evaluate the sensory preference of powdered camel milk compared to fresh milk among pastoralist communities in the Somali Region of Ethiopia.

Methods: **-** Sensoric cross over experiment study was conducted among randomly selected 242 pastoralist communities in Somali Region. Data were collected using structured questionnaires. The sensory trial was single-blinded; participants did not know whether they tasted fresh or powdered camel milk. The sequence was randomized, assigning either fresh/powdered camel milk or powdered/fresh camel milk to each participant. Data was summarized, such as mean, median, frequency, and percentage, and was presented using charts and tables. Bi-variable and Multivariable binary logistic regression identified variables associated with the outcome. Statistical associations were assessed using odds ratios and 95% confidence intervals, with significance declared at a P-value < 0.05. A t-test was used to compare milk spending and milk liking levels between powdered and fresh camel milk.

Results: In this study, 167 (69%; 95% CI: 62.9%–74.5%) preferred fresh camel milk, while 75 (31%; 95% CI: 25.5%–37.1%) preferred reconstituted powdered milk among pastoralists in the Somali Region. Factors significantly associated with preference of reconstituted powdered camel milk included being an urban pastoralist (AOR = 2.02; 95% CI: 1.30, 3.16) and being female (AOR = 1.94; 95% CI: 1.25, 3.01). There is no statistically significant mean difference between fresh camel milk and powdered camel milk regarding willingness to pay.

Conclusion: Although most preferred fresh milk, the mean rating of powder was still high. Powdered milk might be a good alternative in settings where a cool chain is difficult to maintain and shelf life is an issue. Factors such as place of residence and sex of pastoralist significantly influence preference of reconstituted powdered milk. There is no significant difference in willingness to pay between fresh and powdered milk. Focusing on nutritional advantages, safety, and convenience regarding powdered camel milk is crucial for rural and male pastoralists' adoption. Promotional efforts should increase accessibility and practicality without reducing cost.

Key words: Sensory Trial, Camel Milk Powder, Pastoralist Communities, Somali Region, Ethiopia

INTRODUCTION

Camel milk is a vital food source that supplies energy and nutrients to rural communities living in the arid regions of Africa and the Middle East[1, 2]. Additionally, camel milk improves livelihoods and supports economic development both domestically and internationally[3]. Pastoralists in subsistence farming systems keep camels mostly for their milk. They are famous for continuing to produce milk in the face of drought. When milk from cows, lambs, and goats is in short supply during dry seasons and drought years, camels are a very dependable source of milk[4, 5].

Traditional camel milk producers create and consume the product locally. The production process is typically low-tech, and there is minimal global trade in camel milk. Over 80% of the world's camel population is found in Africa, with 60% residing in Eastern African countries[3]. Recent research has made it clear that camel milk, known for its genuine or alleged "medicinal" benefits, is moving beyond the margins. In fact, global camel milk production has seen significant annual growth, surpassing 8% from 2009 to 2019, reflecting the increasing interest in this product[1, 6].

According to statistics on livestock, there are approximately 35.5 million camels worldwide, with the largest populations found in Somalia, Sudan, Niger, Kenya, Chad, Ethiopia, Mali, Mauritania, and Pakistan. In Ethiopia alone, there are about 2.4 million camels, primarily located in the southern and eastern pastoral regions of the country[7, 8].

Conversely, the pronounced seasonality in pastoral environments significantly affects the camel milk subsector. Although camel physiology enables milk production even during dry and drought periods, the overall milk supply tends to decrease. Additionally, during extended dry seasons, herders must travel long distances in search of feed and water[9]. The food industry uses milk powders for many purposes due to their ease of product development, processing, storage, and transportation. Additionally, milk powders are among the best methods for prolonging the shelf life of milk without drastically lowering its quality and nutrients[10, 11].

The advancement of powdered milk production, which is the most effective way to preserve this highly perishable product for later use, has made it possible to include camel milk in the global dairy market. Moreover, since camel milk is often produced in remote areas far from where it is consumed, the only practical method for transporting it in large quantities is by removing its water content, which makes up 88% to 90% of its total weight[12].

Approximately 75,000 tons of camel milk are produced annually in Ethiopia. During the dry seasons, camels continue to produce milk for long periods despite the shortage of pasture. Most of this milk is consumed raw, while a smaller amount is taken in a fermented form. For pastoralists in Ethiopia's Somali region, camel milk plays a vital role in their diet, especially during droughts when milk from cattle and other animals is limited. The milk produced by a lactating camel not only nourishes her calf but also serves as an important nutritional source for people, helping to alleviate food shortages[2, 13].

There are several studies have examined the nutritional benefits and technological processing of camel milk, there is a lack of empirical research on its sensory acceptability in powdered form, especially in pastoralist communities like those in the Somali Region of Ethiopia. No existing studies have specifically assessed the sensory acceptance of camel milk powder among Somali pastoralists. Therefore, conducting a targeted sensory trial is crucial to address this gap and guide the viability and design of future milk powder initiatives in the region.

METHOD AND MATERIALS

Study area and period

The study was conducted in Fafan and Shebelle Zone in Somali Region between February and March 2025. Jigjiga is the capital of the Somali regional state in Ethiopia, situated 630 km from the country's capital, Addis Ababa. As one of the six city council administrations in the region, Jigjiga operates under its own city administration and is divided into 30 kebeles among these, 20 are classified as urban and 10 as rural. The projected population of the city is approximately 182,922 (CSA.2024). Gode town is the capital of Shebelle Zone and it is one of six self-administrated city council. Gode is almost 600KM away from the capital of Somali Regional State Jigjiga and it is the town established along with Shebelle River.

Study design

This study employed Sensoric cross over experiment study design to evaluate the sensory acceptance of camel milk powder among pastoralist communities in the Somali Region. A single-blinded approach was used, in which participants were unaware of whether they were tasting fresh camel milk or reconstituted powdered camel milk.

Source and study population

Source of population

The source population for this study was all pastoralist communities in the Fafan and Shebelle Zones of the Somali region.

Study population

The study population for this study was all randomly selected s all pastoralist communities in Fafan and Shebelle Zones of Somali Region during the study period.

Inclusion and exclusion criteria

Inclusion criteria

The study included pastoralist communities in randomly selected Zones.

Exclusion criteria

All pastoralist communities who are seriously ill, cannot respond, are absent, or refuse to participate in the study were excluded from the study.

Sample size determination

The required sample size of the study participants was determined by using single population proportion formula.

$$n=\frac{\left(Z\frac{\alpha}{2}\right)2\times P(1-P)}{d^2}$$

With following assumptions;

n – The minimum sample size required

P – Estimated proportion of acceptance of powdered camel milk among pastoralist communities.

d – Margin of error 5 %(0.05)

Zα/2- Standard normal value at (1-α) 95% confidence level

Prevalence (P) of acceptance powder camel milk was taken from a study conducted in Kenya, which was 19%[14].

$$\text{Hence, } N = \frac{[Z\alpha 2]^2 p\,(1-p)}{d^2} = \frac{(1.96)^2\,(0.19)(1-0.19)}{(0.05)^2} = 236$$

The final sample, including a 5% non-response rate, was 248.

Sampling technique and procedure

We purposefully selected two zones from the Somali region, ensuring that each zone included both urban and rural pastoralist communities. Based on this urban-rural difference, we allocated our sample proportionally. We then used systematic random sampling to select the pastoralists for our study (**Fig 1**).

***Fig 1**. The flow of the pastoralist communities' selection among residents in Somali region, Eastern Ethiopia,*

Data Collection Method

Data collection was conducted using an interviewer-administered, structured questionnaire designed to capture demographic information and participant responses for each milk sample. The sequence was randomized, assigning either fresh/powdered camel milk or powdered/fresh camel milk to each participant. Each participant was first given 20 ml of camel milk (either fresh or reconstituted) and asked whether they preferred it, responding with either "yes" or "no." They were then provided with water to rinse their mouths before receiving a second 20 ml sample of the other type of camel milk. The acceptability of the second sample was recorded in the same manner.

Study variables

Dependent variable

- Preference powder camel milk

Independent variables

- Place of residence
- Sex of pastoralist
- Age of pastoralist
- Milk expenditure
- Milk liking level

Operational definition

Camel milk powder Preference was defined as the participant's positive response (“yes”) to the question of whether they found the reconstituted camel milk sample acceptable for consumption during a blinded taste test[15].

Milk liking level was assessed using a 5-point Likert scale (1 = Strongly dislike to 5 = Strongly like), following sensory evaluation standards described by Stone and Sidel[16].

Milk spending was assessed by asking participants their willingness to pay for camel milk using a four-point scale, adapted from consumer valuation methods used in food product studies[17].

Data quality control

The quality of data was assured by proper designing and pre-testing (5%) of the questionnaires in those pastoralist communities that were not participating in the study with similar socio-demographic characteristics in order to ensure its validity. Training was given for both data collectors and supervisors by the principal investigator for one day. The sensory trial was single-blinded; participants did not know whether they tasted fresh or powdered camel milk. Both fresh and reconstituted powdered milk samples were prepared using standard procedures (e.g., consistent dilution ratio, serving temperature, and volume: 20 ml per sample). Participants were offered water between samples to cleanse their taste. The training included discussion on the objectives of the study and on the contents of the questionnaire one by one, on procedures, on data collection techniques, and on the issues of the confidentiality of the responses. The collected data was checked by supervisors on a daily basis for any incompleteness, inconsistency, and possible corrections that were made each day based on the identified gaps accordingly. Inconsistencies and possible corrections were made each day based on the identified gaps accordingly.

Data analysis and processing

Data were cleaned, coded, and entered into Kobo Toolbox, then exported to STATA statistical software version 18 for analysis. Descriptive statistics such as mean, median, frequency, and percentage were used and presented in charts and tables. Bi-variable analysis was conducted using binary logistic regression to assess the relative importance of exposure variables in relation to the outcome variable, expressed using odds ratios. In multivariable binary logistic regression, exposure variables with a p-value less than 0.05 and a 95% confidence interval were considered significantly associated with powdered milk acceptance. An independent sample t-test was conducted to assess the mean differences in milk spending between the two types of camel milk (powdered vs. fresh). Additionally, the t-test was used to compare the mean milk liking levels between participants who tasted powdered camel milk and those who tasted fresh camel milk.

Ethical consideration

Ethical was reviewed and approved by the Jigjiga University Research Ethics Review Committee (Approval No. JJU-RERC/002/2025). An official permission letter was also submitted to the relevant administrative offices in the Shebelle and Fafan Zones. Informed, voluntary, written, and signed consent was obtained from the pastoralist communities after the purpose and potential benefits of the study were clearly explained. To ensure participant confidentiality, no identifying information was collected in the questionnaires. Participants were also informed that all data collected would be used solely for research purposes.

Results

Socio-demographic characteristics among pastoralist communities in Somali region, Eastern Ethiopia

A total of 248 pastoralists were selected for the study, of whom 242 participated, resulting in a response rate of 97.6%. The overall mean age of the participants was 34.9 years, with a standard deviation of ±11.4 years. Among male participants, the mean age was 35.9 years (±11.5), while for female participants; it was 33.0 years (±11.1). Two-thirds of the participants, 159 (65.7%), were male. Additionally, more than half of the participants, 132 (54.6%), identified as rural pastoralists, and approximately half, 108 (44.6%), were aged between 30 and 45 years (**Table 1**).

Participants' preference for the fresh or reconstructed milk in Somali Region

In the Somali Region, 167 pastoralists (69%; 95% CI: 62.9%–74.5%) preferred fresh camel milk, while 75 (31%; 95% CI: 25.5%–37.1%) preferred reconstituted powdered camel milk.

Independent Samples T-Test for Comparing Milk Liking Levels Based on Milk Type Preference (Powdered vs. Fresh Camel Milk)

The mean and standard deviation of liking level for fresh camel milk was 4.1(±0.78)**.** The 95% confidence interval indicates that we can be 95% confident that the true mean liking level for fresh camel milk lies between 4.0 and 4.2. The mean and standard deviation of liking level for powdered camel milk was 3.6(±0.94). The confidence interval suggests that the true mean liking level for powdered camel milk is likely between 3.4 and 3.8**.** Pastoralist who preferred fresh camel milk had significantly higher milk liking scores (mean = 4.1) compared to those who preferred powdered camel milk (mean = 3.6). The difference of 0.5 points is statistically significant ($p < 0.001$), and the 95% confidence interval does not include zero, indicating a true difference in liking levels between the fresh camel milk and powdered camel milk (**Table 2**).

Independent Samples T-Test for Comparing Willingness to Pay Based on Milk Type Preference (Powdered vs. Fresh Camel Milk)

The mean and standard deviation of willingness to pay for fresh camel milk 3.0(±0.65). The 95% confidence interval suggests that we can be 95% confident that the true mean willingness to pay for fresh camel milk lies between 2.9 and 3.1.The mean and standard deviation willingness to pay for powdered camel milk is around 2.9(±0.73). The confidence interval indicates that the true mean willingness to pay for powdered camel milk is likely between 2.8 and 3.1. Although pastoralist who preferred fresh camel milk showed a slightly higher mean

willingness to pay (3.0) compared to those who preferred powdered camel milk (2.9), this difference was not statistically significant (p = 0.3136) (**Table 3**).

Bi-variable and multivariable analysis to Preference powder of camel milk

Both bi-variable and multivariable logistic regression analyses were undertaken. These variables included place of Residence, sex and category. Additionally, in the multivariable logistic regression analysis, two variables were found to be significantly associated with Acceptance of powder camel milk.

The odds of preferred powdered camel milk were 2.2 times higher among urban pastoralists compared to rural pastoralists (AOR = 2.2; 95% CI: 1.2 – 4.3). Similarly, female pastoralists had twice the odds of Preferred powdered camel milk compared to male pastoralists (AOR = 2.0; 95% CI: 1.1–3.7) (**Table 4**).

Level of milk liking among pastoralist communities in Somali region, Eastern Ethiopia

Rural pastoralist showed a strong preference for milk, with 58 participants (54.7%) indicating that they "liked" it, while 58 participants (42.7%) from the urban pastoralist also expressed a positive sentiment. Female participants were particularly motivated toward milk, with 44 (50.0%) stating that they "liked" it, and 72 male participants (46.8%) shared the same view. Among age categories, participants aged 30-45 displayed a preference, with 53 (49.1%) stating that they "liked" the milk, while those under 30 showed a neutral response, with 50 participants (54.4%) expressing similar feelings. Fresh camel milk was particularly favored, with 82 participants (49.1%), indicating that they "liked" it, while 34 participants (45.3%) preferred powdered camel milk (**Table 5**).

Willingness to Pay for Camel Milk among Pastoralist Communities in Somali Region, Eastern Ethiopia

In terms of place of residence, the rural pastoralist showed a notable preference, with 65 (61.3%) participants indicating they were willing to pay a fair amount for milk, slightly lower than the urban pastoralist at 90 (66.2%). Regarding sex, female pastoralist demonstrated a stronger willingness, with 60 (68.2%) participants expressing their readiness to pay a fair amount, while 95 (61.7%) male participants reported the same. Among the age categories, those aged under 30 included 62 (67.4%) participants who were willing to pay a fair amount. Regarding milk type, fresh camel milk was favored, with 108 (64.7%) participants indicating they were willing to pay a fair amount, compared to 47 (62.7%) participants for powdered camel milk **(Table 6)**.

Discussion

This finding show that a significantly larger proportion of pastoralists in the Somali Region prefer fresh camel milk compare reconstituted powdered milk. In this study, the preference of reconstituted powdered camel milk among pastoralist communities in Somali Region was 75(31%) with the CI of (25.46%, 37.13%). Our findings are similar to study conducted Nigeria[18]. Powdered camel milk acceptance in both Ethiopia and Nigeria is influenced by their shared pastoralist traditions and dependence on livestock for livelihoods. Large segments of the population in both countries are accustomed to consuming fresh milk, which contributes to only moderate preferred of powdered alternatives. Additionally, they encounter comparable obstacles in promoting powdered milk, including low awareness of its nutritional value and differing views on product quality.

On the other hand, the acceptance of reconstituted powdered camel milk in this study was lower than in a study conducted in Kenya[19]. This difference may be attributed to cultural norms, economic conditions, and more advanced market development in Kenya. A better developed dairy sector and increased public awareness of powdered milk, bolstered by effective marketing and consumer education initiatives, are advantageous for Kenya. Its popularity is hindered in the Somali Region by inadequate infrastructure, a cultural preference for fresh milk, and a lack of knowledge about the advantages of powdered milk.

Place of residence is significant associated with acceptance of reconstituted powdered camel milk. Urban pastoralists were 2.2 times higher to accept powdered camel milk compared to rural pastoralists (AOR = 2.2; 95% CI: 1.17–4.28). This study consisted the study conducted Comparative Analysis of Acceptability of Camel Milk among Rural and Urban Consumers in Kenya[20]. Urban populations tend to have better access to information and resources, which helps people, become more aware of the nutritional advantages of powdered milk. The discrepancy may be due to the broader availability of powdered milk products in urban markets, where effective marketing strategies and better consumer exposure encourage their acceptance.

Sex of the pastoralist was significant associated with acceptance of reconstituted powdered camel milk. Female pastoralists had twice the odds of accepting powdered camel milk compared to male pastoralists (AOR = 2.0; 95% CI: 1.09–3.66). This possible may be because women are more likely to manage the food resources in the home and are more willing to provide their families with a variety of dietary options. Comparatively speaking to males,

women are more likely to accept powdered camel milk because they are more proactive in embracing innovations that can improve household welfare.

This study show that, liking levels between the fresh camel milk and powdered camel milk are not same. This finding is consistent the study conducted in European and Mediterranean Countries[21]. The taste and apparent freshness of fresh camel milk make it the preferred option above its powdered equivalent. Associating fresh camel milk with superior flavor, participants in sensory evaluations frequently expressed a stronger preference for it.

There is no significant difference mean willingness to pay between fresh camel milk and fresh camel milk, this difference was not statistically significant ($p = 0.3136$). This finding line with the study conducted Aba'ala woreda, Afar Region[22]. This might other elements, such taste preference, familiarity, accessibility, or perceptions of quality, can have a greater impact on consumer choice than price. Given that customers seem to be equally willing to pay for either product when price is the only consideration, interventions meant to increase the adoption of powdered camel milk should concentrate on addressing these non-price aspects.

Conclusion

Although the majority preferred fresh camel milk, the average rating for powdered milk remained high. This suggests that powdered milk could serve as a suitable alternative in settings where maintaining a cold chain is challenging and shelf life is a concern. Place of residence and sex of pastoralist are significantly associated with preference of powder camel milk. While participants showed a significant preference for fresh camel milk over powdered camel milk in terms of liking, the difference in their willingness to pay for the two types was not statistically significant. Implementing focused educational efforts that highlight the product's nutritional advantages for men is crucial. The nutritional advantages, safety, and convenience of powdered camel milk should be highlighted in focused awareness and education initiatives to increase rural pastoralists' adoption of the powdered camel milk. Promotional efforts for powdered camel milk should concentrate on increasing its accessibility and practicality without reducing its cost.

Author contributions

All authors participated to the study's beginning, design, data collection, analysis, and interpretation. During paper submission, the writers critically reviewed the work and

contributed to its progress. The authors all agreed to accept responsibility for the content of the work after reading and approving the final version of the work.

Conflict of interest

The authors sustain no conflict of interest.

Funding statement

The authors did not receive any support for this study.

Data availability statement

If you make a legitimate request, the relevant author will provide you with the data supporting the results of this study.

ORCID ID

Ahmed Mohammed Ibrahim https://orcid.org/0000-0002-3493-8473.

Reference

1. Gader AGMA and Alhaider AA. The unique medicinal properties of camel products: A review of the scientific evidence. *Journal of taibah university medical sciences* 2016; 11: 98-103.
2. Berhe T, Seifu E and Kurtu MY. Physicochemical properties of butter made from camel milk. *International Dairy Journal* 2013; 31: 51-54.
3. Faye B, Madani H and El-Rouili SA. Camel milk value chain in Northern Saudi Arabia. 2014.
4. Alhadrami G and Faye B. Camel. 2022.
5. Farah Z. Milk| Camel Milk. 2011.
6. Kuczuk A and Widera K. Proposed Changes in Polish Agricultural Products Consumption Structure for 2030 Based on Data from 2008–2018. Sustainability 2021, 13, 7536. s Note: MDPI stays neutral with regard to jurisdictional claims in published ..., 2021.
7. Faye B. How many large camelids in the world? A synthetic analysis of the world camel demographic changes. *Pastoralism* 2020; 10: 25.
8. Prakash A and Stigler M. Fao Statistical Yearbook. Food and Agriculture Organization of the United Nations. 2012.
9. Galmessa U and Fita L. Dairy development in Ethiopia: Present scenarios, challenges and future prospects for development. 2021.
10. Sharma A, Jana AH and Chavan RS. Functionality of milk powders and milk-based powders for end use applications—a review. *Comprehensive reviews in food science and food safety* 2012; 11: 518-528.
11. Pugliese A, Cabassi G, Chiavaro E, et al. Physical characterization of whole and skim dried milk powders. *Journal of Food Science and Technology* 2017; 54: 3433-3442.
12. Konuspayeva G and Faye B. Recent advances in camel milk processing. *Animals* 2021; 11: 1045.
13. Olusoga KO. *Cost-Benefit Analysis of Powdered Camel Milk Production in the Somali Region of Ethiopia*. Eastern Mediterranean University (EMU)-Doğu Akdeniz Üniversitesi (DAÜ), 2021.
14. Akweya B, Gitao C and Okoth M. The acceptability of camel milk and milk products from north eastern province in some urban areas of Kenya. *African Journal of Food Science* 2012; 6: 465-473.
15. Al Kanhal HA. Compositional, technological and nutritional aspects of dromedary camel milk. *International Dairy Journal* 2010; 20: 811-821.

16. Stone H, Bleibaum RN and Thomas HA. *Sensory evaluation practices*. Academic press, 2020.
17. Lusk JL and Hudson D. Willingness-to-pay estimates and their relevance to agribusiness decision making. *Applied Economic Perspectives and Policy* 2004; 26: 152-169.
18. Adewumi O, Lawal-Adebowale O and Adegbemile D. Assessment of farm families acceptability of small ruminants' milk for consumption in selected rural communities in Ogun state, Nigeria. *Journal of Agricultural Extension and Rural Development* 2015; 7: 135-141.
19. Kashongwe BO. *Assessing farm level practices affecting milk production, quality and postharvest losses in smallholder dairy and pastoral camel herds of Kenya*. Egerton University, 2017.
20. Emukule J, Ngigi M, Guliye A, et al. Comparative Analysis Of Acceptability Of Camel Milk Among Rural And Urban Consumers In Kenya. 2011.
21. Profeta A, Enneking U, Claret A, et al. Consumer acceptance and preference for camel milk in selected european and mediterranean countries. Sustainability, 14 (22), 15424. 2022.
22. Halefom YN. *Households Willingness to Pay for Camel Milk in Aba'ala Woreda, Afar Regional State*. The Institute of Development Studies and Partner Organisations, 2014.

Table 1: - Socio-demographic characteristics among pastoralist communities in Somali region, Eastern Ethiopia

**Socio-demographic variables**	**Categories**	**Frequency**	**Percent**
Pace of residence	Rural	106	43.8
	Urban	136	56.2
Sex of participant	Male	154	63.6
	Female	88	36.4
Age group	<30 years	92	38.0
	30 - 45 years	108	44.6
	>45 years	42	17.4

Table 2: - Independent Samples T-Test for Comparing Milk Liking Levels Based on Milk Type Preference (Powdered vs. Fresh Camel Milk) in Somali Region. Ethiopia

Group	Observation	Mean	Standard error	Standard deviation	95% Confidence interval
Fresh camel Milk	167	4.1	0.06	0.78	(4.0, 4.2)
Powder camel	75	3.6	0.11	0.94	(3.4, 3.8)
Combined	242	4.0	0.06	0.86	(3.8, 4.1)
Difference		0.5	0.12		(0.3, 0.8)

Table 3: - Independent Samples T-Test for Comparing Willingness to Pay Based on Milk Type Preference (Powdered vs. Fresh Camel Milk) in Somali Region, Ethiopia

Group	Observation	Mean	Standard error	Standard deviation	95% Confidence interval
Fresh camel Milk	167	3.0	0.05	0.65	(2.9, 3.1)
Powder camel	75	2.9	0.09	0.73	(2.8, 3.1)
Combined	242	3.0	0.04	0.68	(2.9, 3.1)
Difference		0.1	0.09		(-0.1, 0.3)

Table 4: - Factor associated with acceptance of reconstituted powdered camel milk in Somali Region

**Variables**	**Category**	**Acceptance powder camel Milk**		**Bi-variable analysis**		**Multi-variable analysis**	
		**Yes N (%)**	**No N (%)**	**COR (95% CI)**	**P-value**	**AOR (95% CI)**	**P-value**
Place Residence	Rural	21(19.8)	85(80.2)	1	0.001	1	0.015
	Urban	54(39.7)	82(60.3)	2.7(1.5, 4.1)		2.2(1.2, 4.3)	
Sex	Male	36(23.4)	118(76.6)	1	0.001	1	0.024
	Female	39(44.3)	49(55.7)	2.6(1.6, 4.6)		2.0(1.1, 3.7)	
Age category	<30 years	30(32.6)	62(67.4)	1.2(0.5, 2.7)	0.640	1.1(0.5, 2.7)	0.682
	30 – 45 years	33(30.6)	75(69.4)	1.1(0.5, 2.4)	0.812	1.3(0.6, 3.0)	0.540
	>45 years	12(28.6)	30(71.4)	1		1	

Table 5: - Preferences for Milk Among pastoralist communities in Shebelle and Fafan Zone, Somali region, Eastern Ethiopia

**Variable**	**Category**	**Number**	**Strongly dislike**	**Dislike**	**Neutral**	**Like**	**Strongly like**
Pace of residence	Rural	106	0(0.0%)	3(2.8%)	14(13.2%)	58(54.7%)	31(29.3%)
	Urban	136	3(2.2%)	7(5.1%)	33(24.3%)	58(42.7%)	35(25.7%)
Sex of participant	Male	154	1(0.7%)	7(4.6%)	24(15.6%)	72(46.8%)	50(32.5%)
	Female	88	2(2.3%)	3(3.4%)	23(26.1%)	44(50.0%)	16(18.2%)
	<30	92	0(0.0%)	0(0.0%)	21(22.8%)	50(54.4%)	21(22.8%)

Age group	30 – 45	108	1(0.9%)	7(6.5%)	20(18.5%)	53(49.1%)	27(25.0%)
	>45	42	2(4.8%)	3(7.1%)	6(14.3%)	13(31.0%)	18(42.9%)
Milk type	Fresh camel Milk	167	1(0.6%)	3(1.8%)	26(15.6%)	82(49.1%)	55(32.9%)
	Powder camel milk	75	2(2.67%)	7(9.3%)	21(28.0%)	34(45.3%)	11(14.8%)

Table 6: - Willingness to Pay for Milk Among pastoralist communities in Somali region, Eastern Ethiopia

Variable	Category	Number	not willing to pay	A little amount	Fair amount	Premium Amount
Pace of residence	Rural	106	1(0.9%)	19(17.9%)	65(61.3%)	21(19.8%)
	Urban	136	7(5.2%)	11(8.0%)	90(66.2%)	28(20.6%)
Sex of participant	Male	154	6(3.9%)	20(12.9%)	95(61.7%)	33(21.4%)
	Female	88	2(3.2%)	10(11.4%)	60(68.2%)	16(18.2%)
Age group	<30	92	--	15(16.3%)	62(67.4%)	15(16.3%)
	30 – 45	108	5(4.6%)	10(9.3%)	67(62.0%)	26(24.1%)
	>45	42	3(7.1%)	5(11.9%)	26(61.9%)	8(19.0%)
Milk type	Fresh camel Milk	167	4(2.4%)	20(12.0%)	108(64.7%)	35(21.0%)
	Powder camel	75	4(5.3%)	10(13.3%)	47(62.7%)	14(18.7%)

Figure

Click here to access/download;Figure;Fig 1.jpg

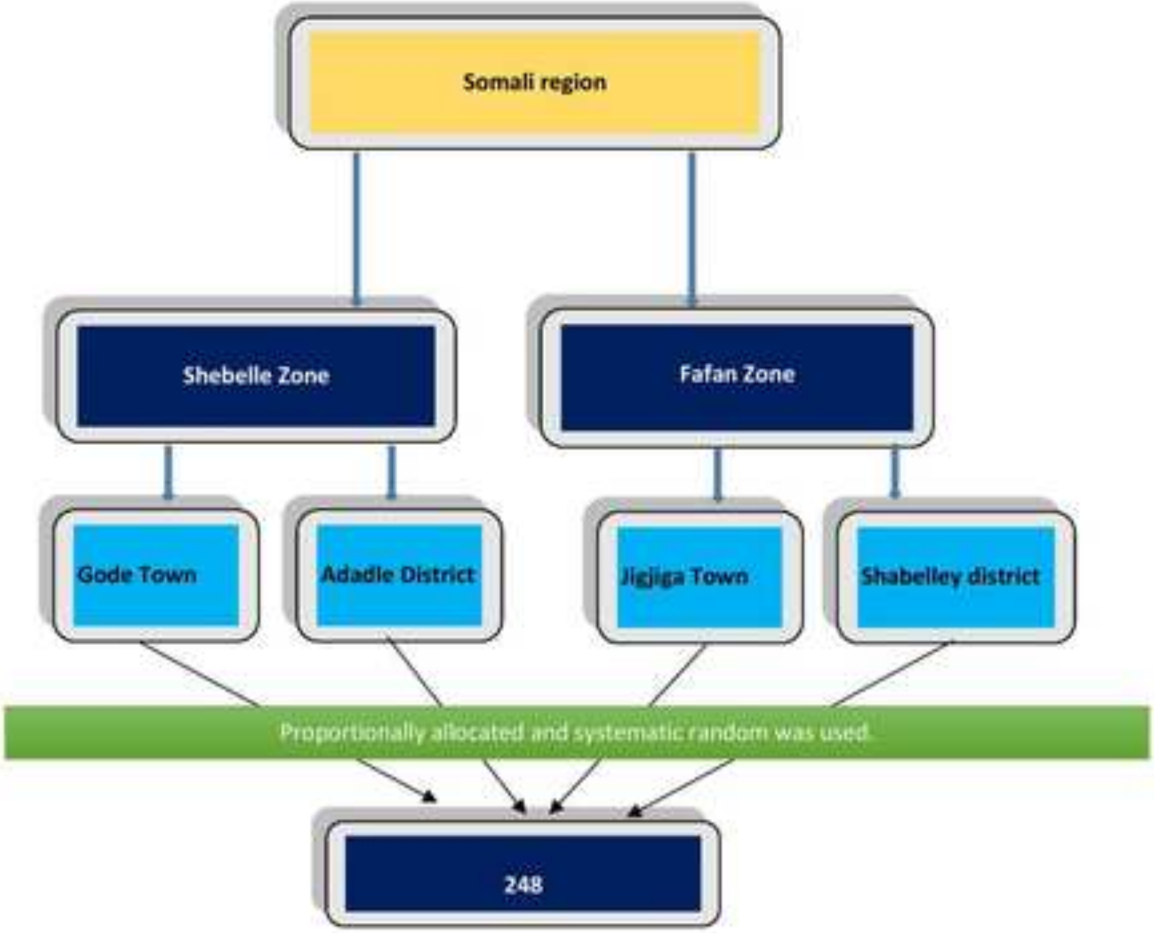

Fig 1. The flow of the pastoralist communities' selection among residents in Somali region, Eastern Ethiopia.

Click here to access/download
Supporting Information
ANNEX.docx
