# Supplementary material for: Sensory trial of camel milk powder among pastoralist communities of the Somali Region, Ethiopia
Source: PLoS One. 2025 Oct 30;20(10):e0333358. doi: 10.1371/journal.pone.0333358 (PMC12574850; doi:10.1371/journal.pone.0333358)
Supplement: Attachment — Submitted filename: Response_to_Reviewers_auresp_2.docx [file pone.0333358.s007.docx]

**Reviewer Comments to the Author**

Reviewer #2: -

- Consider integrating the “Limitations” paragraph directly into the end of the Discussion section as a final subsection.
- Following PLOS ONE formatting guidelines, keep the full figure title and description in the figure caption only. “Figure legends”

**Authors' Responses**

- Dear reviewer, Thank you for your valuable comments: we incorporated and integrated the “Limitations” paragraph directly into the end of the Discussion section as a final subsection as you suggested.
- We appreciate your suggestion. By following PLOS ONE formatting guidelines, we kept the full figure title and description in the figure caption only. “Figure legends”
